# Is knee osteoarthritis a symmetrical disease? Analysis of a 12 year prospective cohort study

**DOI:** 10.1186/1471-2474-13-153

**Published:** 2012-08-22

**Authors:** Andrew J Metcalfe, Maria LE Andersson, Rhian Goodfellow, Carina A Thorstensson

**Affiliations:** 1 School of Medicine, Cardiff University, Cardiff CF10 3XQ, UK; 2 Research and Development Centre, Spenshult, Oskarström, Sweden; 3 Department of Trauma and Orthopaedics, University Hospital of Wales, Heath Park, Cardiff CF14 4XW, UK

**Keywords:** Osteoarthritis, Bilateral, Unilateral, Cohort

## Abstract

**Background:**

The aim of this study was to document the development of bilateral knee osteoarthritis over a 12 year period using a middle-aged population-based cohort with knee pain at inclusion.

**Methods:**

One hundred and forty three patients aged 35 to 54 were recruited from a population based cohort of 279 subjects who had knee pain at baseline and assessed with clinical and radiographic data, with 5 and 12 year follow up. The data was analysed with regard to the development and progression of uni- and bilateral knee osteoarthritis over 12 years. A definition of KL = 1 was used to define radiographic disease.

**Results:**

24 of the 30 (80%) patients with unilateral disease at baseline developed bilateral disease after 12 years. At baseline 37 patients (26%) had bilateral disease, whereas that number increased to 65 (52%) at 5 years and 100 (70%) at the 12 year follow up. The most common pattern was medial compartment involvement in both knees. Six patients had lateral compartment disease in one knee and medial in the other whereas only two had lateral compartment disease bilaterally.

**Conclusions:**

Bilateral knee osteoarthritis is very common with time, as the majority of sufferers will eventually develop radiographic disease in both knees. Clinicians need to be aware of the ‘joint at risk’ and researchers need to remember to account for both knees when assessing the relationship between physical function, pain and structural disease. The other knee should not be used for comparison, even if it appears to be normal at baseline.

## Background

Knee osteoarthritis was historically considered an ‘asymmetric’ disease and most research continues to focus on each joint as a single entity. Cross sectional studies have shown that bilateral knee pain is a frequent problem in the community [1-3]. Each additional joint affected by osteoarthritis results in a decrease in physical function and an increase in overall pain [1-3]. A recent study demonstrated that bilateral knee pain was an independent risk factor for poor physical function [4]. However, there have been very few studies which have addressed the prevalence or natural history of bilateral disease radiologically.

Whereas joint injury (bony or soft tissue) usually affects one joint alone, there are many reasons why knee osteoarthritis would tend to progress to bilateral disease. Genetic influences and inherent mal-alignment would be expected to lead to bilateral disease [5,6]. A recent gait analysis study found abnormal loading in the unaffected knee of patients with unilateral knee osteoarthritis, implying that patients with a painful joint may accelerate disease in other joints due to changes in gait [7].

We do know that bilateral knee osteoarthritis is particularly common in people with advanced disease, with a previous study finding that eighty-seven percent of patients awaiting total knee replacement (TKR) have radiological evidence of osteoarthritis on the other side [8]. Despite this information, we do not know the timescales involved in this process, or whether this is a common problem in community arthritis sufferers, or just the subset who develop disease severe enough to require arthroplasty.

There is a shortage of longitudinal studies examining the natural history of bilateral knee osteoarthritis in the literature, and timing of progression to bilateral disease has not been clarified. In a previous study with 2 year follow-up, 34% of patients with unilateral disease subsequently developed disease in the contra-lateral knee, however follow up was relatively short and the study was restricted to females only [9].

It would appear that community studies of osteoarthritis find lower rates of bilateral disease than studies performed at the time of joint arthroplasty, implying that bilateral disease becomes more common with time [1-3,8,9]. However, temporal data is absent and definitions of symptomatic or structural osteoarthritis vary widely between studies. Also, we do not how representative arthroplasty patients are of community-recruited osteoarthritis sufferers, given that those seeking secondary care treatment form a small proportion of community osteoarthritis sufferers [10].

A longitudinal cohort study with community recruitment has a number of advantages for understanding the development of bilateral disease as it allows us to document the change over a set time period, using a standard definition of disease made by the same observers.

This approach allows researchers, clinicians and patients to understand the disease process and the risk of further disease in the future over a known time period. Researchers wishing to plan longitudinal studies need to be able to quantify the changes that would be expected on the other side, as the development of bilateral disease has an impact on gait and on overall function, therefore affecting the results of research [4,11]. Patients frequently ask about the risk of new disease on the contra lateral side and increased knowledge about prognosis for their condition might allow clinicians to offer more specific advice, and to understand the potential benefits of preventative treatments to protect an apparently normal joint on the other side (such as footwear modification, gait retraining, or simply lifestyle advice).

The focus of this study was to determine whether knee osteoarthritis primarily affects both knees over time. The primary aim of this study was therefore to describe the development of bilateral knee osteoarthritis, as opposed to unilateral disease, over a 12 year period using a middle-aged population-based cohort with knee pain at inclusion. A secondary aim of the study was to explore whether the development of bilateral knee OA was related to age, gender, baseline knee pain or body mass index.

## Methods

**Figure 1:**
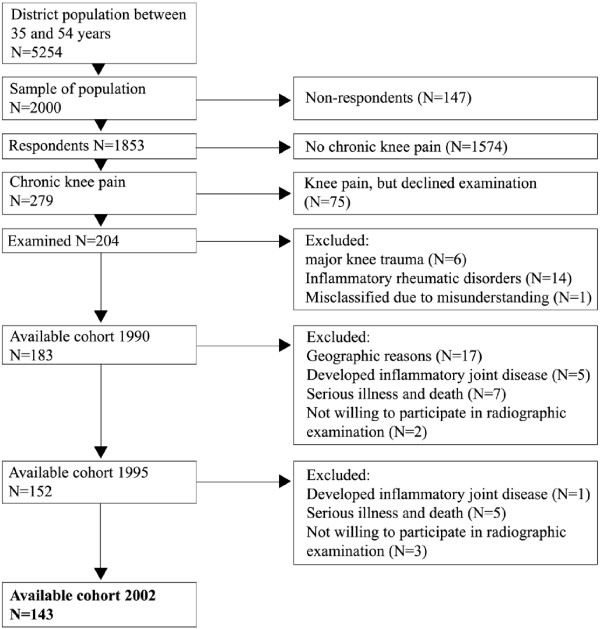
**Flowchart of recruitment for the study (from Thorstensson et al. 2009[**[7]**]).**

Data was extracted from an ongoing prospective study using a population-based cohort, the Spenshult cohort, to explore the natural course of knee osteoarthritis [12,13]. In brief, inclusion criteria were age 35–54 years, chronic knee pain (pain on most days for 3 months) and no history of previous knee injury or inflammatory joint disease. Bilateral radiographic knee examination were done at baseline, five and twelve years follow up. The detailed procedure has been described previously and the full cohort was analysed for the purposes of this paper [12,13]. The primary time point of interest in this study was 12 years. Two hundred and four patients were included at baseline with 143 (63 female and 80 male) having had radiographs of both knees taken at baseline and 12 year follow up, giving a follow up rate of 78% (Figure 1). Eighteen patients out of the 143 did not have radiographic examination at the 5-year follow up time point.

The condition of the patella-femoral joint was not assessed at baseline and so was not included in the analysis. Therefore, only the tibio-femoral data was examined for the purposes of this paper. The Kellgren-Lawrence (KL) classification system was used to assess the severity of osteoarthritis [14,15]. All radiographs were assessed by the same experienced radiologist blind to the patient details. Radiographs from baseline and follow ups were evaluated on different occasions. Inter- and intra- observer agreement for reading of radiographs has been described previously and found to be high (κ = 0.72-0.98) [12,16,17].

Previous studies in Spenshult and elsewhere have found that KL grade 1 represents a genuine early stage in knee osteoarthritis, with detectable cartilage deficits on MRI and likely to progress to more severe disease [13,16,18]. The use of KL 2 as the definition of radiographic OA has been preferred by some authors whilst others have found it underestimates the prevalence of disease in many cases [13,16,18,19]. Both definitions depend on the radiographic protocol used and have considerable inter-observer variability [20,21]. In the Spenshult-cohort KL grade 1 has previously been used as a primary definition of radiographic tibio-femoral osteoarthritis and was also the primary definition used in this study [13,16]. A secondary definition of KL grade 2 was also recorded to aid interpretation of the data.

In order to determine which compartment was most affected, measures of joint space width were taken from the medial and lateral compartments using a ruler. At the 5 year and 12 year follow up period, minimum joint space widths were recorded and subsequently expressed as a categorical joint space width score between 0 and 3, according to the OARSI-OMERACT taskforce report from 2008 [21]. A score above 0 in either compartment defined the presence of osteoarthritis, and these findings are reported in the results as a secondary definition of unilateral or bilateral OA.

The scores for medial and lateral compartments in each joint were examined relative to each other to determine the most affected compartment. A higher score in the medial compartment was classified as representing predominantly medial disease, a higher score in the lateral compartment was classified as representing predominantly lateral disease, equal scores in both compartments were recorded as equal disease in both compartments, and a proportion of patients were found to have no joint space narrowing.

Ninety-five percent binomial confidence intervals (CI) were calculated for proportions, and relative risks were calculated for between group comparisons.

Height and weight were measured at the study start and body mass index (BMI) calculated. Subjects were asked at the start of the study if they had ‘pain in any of your knees practically daily for the last three months’ and were only included in the cohort if they answered yes. They were subsequently seen by a rheumatologist at the entry to the study, who determined whether the pain was in the right knee, the left knee or both knees. The full protocol for this has been published previously [12,13].

Logistic regression was used to study the effect of age, gender, BMI and baseline pain (unilateral vs. bilateral knee pain) on the likelihood of developing bilateral as opposed to unilateral disease over 12 years. In order to understand the effects of these factors on new onset bilateral disease over 12 years (as opposed to prior to the start of the study), patients with pre-existing bilateral disease at baseline (n = 37) were excluded from the regression analysis. A cut off of BMI > 30 kg/m^2^ was used to determine obesity [22]. Statistics was performed using SPSS version 16 (SPSS Inc., Chicago, Illanois, USA).

## Results

The age, gender, BMI, unilateral/bilateral pain and radiographic severity of the group at baseline is given in Table 1. There was no gender difference in terms of age or body mass index at baseline in this cohort (mean age for males 44.8 (SD 5.9) and females 44.8 (SD 5.9), p = 0.95; mean BMI for males 26.2 (SD 3.3) and females 26.2 (SD 4.7) p = 0.94).

**Table 1:** Demographics of the study group at baseline divided by gender

	**Male**	**Female**	**Total**
Numbers of subjects	80	63	143
Mean Age	44.8 (5.8)	44.8 (5.9)	44.8 (5.9)
Mean Body Mass Index (kg/m^2^)	26.2 (4.6)	26.2 (3.2)	26.2 (3.9)
Pain at baseline (number of subjects)			
Unilateral	40	24	64
Bilateral	40	39	79
KL grade at baseline (all knees, n = 286)			
0	99	83	182
1	45	28	73
2	10	4	14
3	6	11	17
4	0	0	0

Age and body mass index are reported as mean (standard deviation).

**Figure 2:**
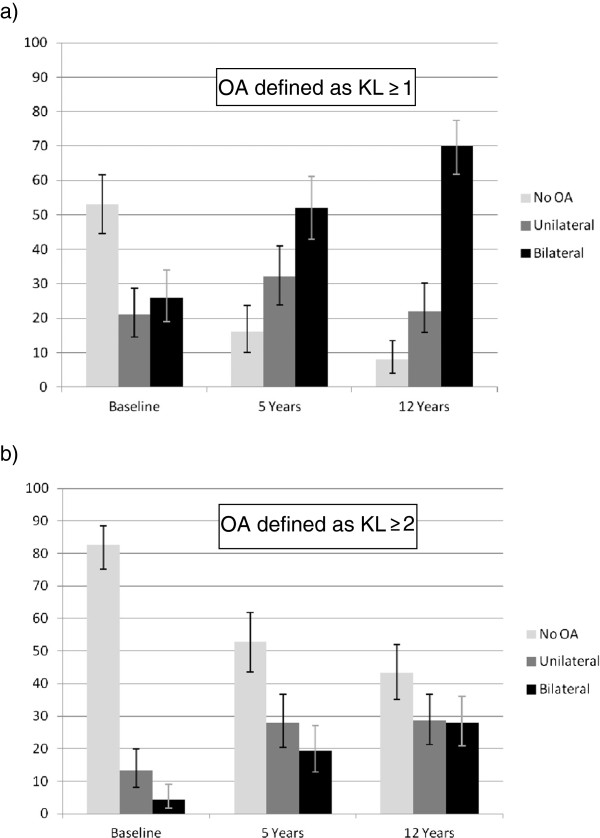
**Percentage of subjects with no radiographic change, unilateral and bilateral changes at each time point. **Error bars represent 95% confidence intervals. The term ‘No OA’ refers to subjects with no evidence of disease in either knee. **a**) using a definition of KL ≥ 1 for osteoarthritis. **b**) using a definition of KL ≥ 2 for osteoarthritis.

At baseline, 76 out of 143 (53%) participants had no changes on x-ray, and 37 (26%) had changes in both knees (Figure 2). At 5 and 12 years the number with bilateral disease had increased to 65 (52%) and 100 (70%) respectively. Of the 18 patients who were not seen at 5 year follow up, 12 had no changes on x-ray at baseline, 4 had unilateral changes and 2 had bilateral changes. At the 12 year follow up, 4 of the 18 had unilateral changes and 14 of the 18 had bilateral disease.

Of those who started the study with knee pain but no radiological changes, 23/76 (30% (95%CI 20.3 to 41.9%)) developed unilateral changes after 12 years, whereas 41/76 (54% (95%CI 42.1 to 65.4%)) developed bilateral changes. Twenty-four of the 30 (80% (95%CI 61.4 to 92.3%)) patients with unilateral disease at baseline developed bilateral disease after 12 years.

If a definition of osteoarthritis as KL ≥ 2 was preferred, then at baseline 118 (83%) patients had no osteoarthritis, 19 (13%) had unilateral osteoarthritis and 6 (4%) had bilateral osteoarthritis. Using the same definition, at 5 years there were 35 (28%) with unilateral osteoarthritis and 24 (19%) with bilateral osteoarthritis. At 12 years there were 41 (29%) with unilateral osteoarthritis and 40 (28%) with bilateral osteoarthritis. Of those who had unilateral KL ≥ 2 disease at baseline, 12/19 (63%) subsequently developed bilateral KL ≥ 2 osteoarthritis.

**Figure 3:**
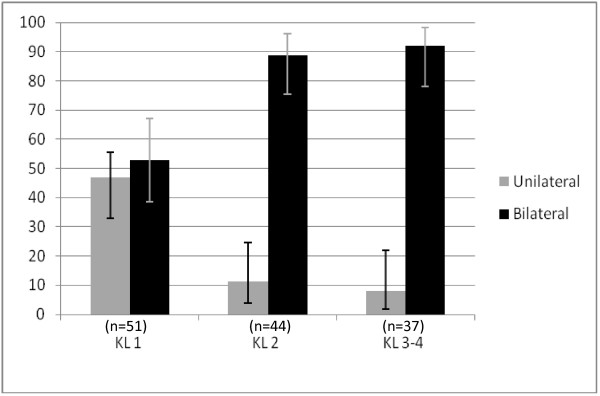
**Percentage of subjects with unilateral and bilateral osteoarthritis at 12 year follow up, classified according to the Kellgren Lawrence grade of the most severely affected knee. **Error bars represent 95% confidence intervals.

There was an increasing association between radiological severity in the most affected knee and bilateral changes (Figure 3). At the 12 year follow up, of the patients whose most severe knee was KL grade 1, 27/51 (53%) had bilateral disease, whereas 39/44 (89%) of those with KL grade 2 and 34/37 (92%) of those with KL grade 3 and above had bilateral involvement.

Using the OARSI-OMERACT joint space width scoring to classify osteoarthritis, 79 patients (63%) had bilateral disease at 5 year follow up, and 106 patients (74%) with bilateral disease at 12 year follow up.

Of those patients with bilateral disease at 12 year follow up, 73 patients had predominantly medial compartment disease in both knees, 6 patients had medial compartment disease in one knee and lateral compartment disease in the other knee whereas only 2 had predominantly lateral disease in both knees. Twenty-five patients had equal scores in the medial and lateral compartment of at least one knee.

At baseline, bilateral pain was reported by 48 of the 76 patients with no x-ray changes, 13 of the 30 patients with unilateral radiographic change, and 18 of the 37 patients with bilateral x-ray changes.

Age, gender, BMI > 30 or bilateral knee pain at baseline had no influence on the development of bilateral as opposed to unilateral disease (For gender odds ratio (OR) 1.19 [95%CI 0.53-2.67], p = 0.70; for BMI > 30 OR 1.09 [95%CI 0.35-3.38], p = 0.92; for bilateral knee pain at baseline OR 1.48 [95% CI 0.69-3.55], p = 0.33; and mean age without developing bilateral disease 43.8 years, mean age in those who developed bilateral disease 44.8 years, p = 0.41). There was no statistical difference in age, gender or BMI between those with and without bilateral disease at baseline (data not shown).

## Discussion

In this population based, middle-aged cohort a majority had early disease (i.e. joint pain with no or minor radiographic changes) at baseline. Despite this, 70% had bilateral radiographic changes 12 years later. Of those with unilateral knee osteoarthritis at baseline, 80% developed bilateral disease over 12 years. Osteoarthritis may have an asymmetrical onset but it has a tendency to affect both joints with time.

We have previously suggested that knee pain is the first sign of knee osteoarthritis [13], and the results from the present study add to this message that early knee osteoarthritis, without any known previous injuries to the anterior cruciate ligament or the meniscus, in most cases develops into a bilateral disease. We are unable to comment on the cause for the pattern of osteoarthritis progression that we have observed in this paper, as this would require much more complicated biological and biomechanical methods, however at present such studies are not available with the length of follow up achieved in our cohort.

As patients with joint injury were excluded from this cohort, it would be interesting to study a ‘unilateral joint injury’ cohort in the future, to see if those individuals who developed unilateral disease as a result of injury also progressed towards bilateral disease in the same way. Whilst it would be easy to assume that our results are solely due to genetic pre-disposition, changes in gait, mechanical loading and behaviour also need to be considered as causes for bilateral disease development [7]. A comparative cohort study which assessed those with knee injury at baseline and those without a known cause would be of interest to understand this question in more detail.

Medial compartment osteoarthritis was the most common finding at 12 years, and this is very much expected. However, it was interesting to find that even amongst patients with lateral compartment changes in one knee, medial compartment disease was more common in the other knee. Numbers were too low to examine statistically and would need to be confirmed in larger cohorts, but this may be an interesting sub-group to study in the future.

In a previous study with 2 year follow-up, 34% of patients with unilateral disease subsequently developed disease in the contra-lateral knee (with osteoarthritis defined as KL ≥ 2) which corresponds reasonably well with our longer-term results [9]. It may be argued that the contra-lateral knee is not necessarily free of disease in patients with unilateral radiographic disease using standard radiographic protocols [20]. However, the development of incident radiographic osteoarthritis in this joint does demonstrate significant progression of the disease process and therefore remains a relevant end point.

It can be seen from Figure 3 that bilateral knee osteoarthritis becomes much more common as the disease severity increases. This is no surprise, but it is important to note that this happens at an early stage in the disease process, as those with KL = 2 in one knee were very likely to have disease present on the other side. Preventive interventions should therefore be instituted early in order to avoid or postpone symptomatic and radiographic knee osteoarthritis on the contra lateral side.

The variable relationship between pain and radiographic disease is well documented [13,23,24]. However, more severe radiographic osteoarthritis is associated with increased frequency and intensity of pain, as well as reduced physical function [24,25]. The major focus on radiographic disease in this study is therefore clinically relevant, as bilateral structural disease progression would be expected to result in increased pain and functional decline over time. Pain is not necessarily the most important symptom to the patients, but the impact of pain on physical function. It has previously been shown that impaired physical function has more impact on help seeking than pain severity [26]. Although all patients had chronic pain at inclusion, a larger sample size would be required to reliably examine the relationship between laterality, severity and the presence or absence of pain at follow-up.

The lack of association between gender, age or obesity and bilateral osteoarthritis needs to be interpreted with caution, since the current study did not have sufficient numbers to separate out the effects of these factors properly. Whilst the sample size used in this study was good overall, some of the numbers in individual cells were relatively small and this may have been a factor in the lack of a significant result. This deficiency is emphasised by the width of the confidence intervals for the odds ratios, which were large. However, the odds ratios were close to 1 and differences in means were small, which suggests that even if these factors were significant in a large study, they may not have clinically relevant effects. The higher odds ratio (OR = 1.49) for bilateral pain at baseline suggests that this may be a risk factor for bilateral disease. Larger studies would be required to analyse this in more detail.

The primary time-point of interest was the 12 year follow-up. The loss to follow up at 5 years (18/143) was relatively small and the results between baseline and 12 year follow up for those 18 patients were representative of the results for the whole population. We therefore believe that it is unlikely that the loss of those patients at the 5 year time point had a significant effect on the 5 year findings.

As this was a population based cohort that would traditionally be considered at low risk for osteoarthritis, with a high proportion of males, low levels of obesity and a low age range compared to most osteoarthritis population studies, the high incidence of bilateral radiographic disease after 12 years was striking. Knee pain cannot be considered innocent, even in a young and relatively healthy group of patients.

The gender distribution is not typical of a cohort of patients with established osteoarthritis. Rather, the gender distribution was representative of the community that the patients were recruited from at the start of the study [12].

The reason for the relatively high number of males may lie in the age group of the patients. The gender distribution in this study is consistent with other studies which have examined middle-aged subjects with knee osteoarthritis, as the proportion of males with knee osteoarthritis tends to be higher in younger age groups [27,28]. We believe that the findings are generalisable to middle-aged patients who present with knee pain in the community, which was the purpose of the study when it was initiated.

Therefore the weaknesses of the study are the relatively small sample size, particularly when subgroups were considered or in the logical regression results. The logistic regression analysis was performed on both patients with no disease at baseline and those with unilateral disease at baseline and we accept that these may be two distinct phenotypes which progress differently towards bilateral disease, although we have seen no evidence to either confirm or refute this assertion. The lack of association between bilateral disease and age, gender, BMI and pain should therefore be considered with some caution.

Certainly, more information would have been gained from yearly radiographs compared to the two time points of follow-up in the study. Whilst this might have been preferable scientifically, it would require a much large research budget, and compliance would be a challenge, as well as the ethical issues of radiation doses and inconvenience to the participants. Yearly radiographic examination might not have influenced the overall conclusion, namely that bilateral osteoarthritis is common in this population over a prolonged period of time.

The strengths of the study were that this was a prospectively collected, community based dataset with radiographic data collected over a long time period and at two time points. We believe that numbers were more than adequate to draw the primary conclusion of the paper, that bilateral disease is a common outcome in patients who present with knee pain and particularly in those who already have evidence of osteoarthritis in one knee.

A recent analysis of a large longitudinal cohort of patients at high risk of osteoarthritis found that bilateral knee pain was an independent risk factor for poor physical function, even when pain severity was accounted for [4]. The authors of this paper speculated that this may be due to the loss of a ‘good limb’ to compensate during functional activities. Given the high frequency of bilateral disease after 12 years in our study, it is likely that the development of disease in a second joint is a significant cause of additional disability in the population.

Studies have demonstrated that the biomechanics of the unaffected limb are not normal in patients with unilateral knee osteoarthritis, and also that gait asymmetries exist in patients with unilateral osteoarthritis that subsequently change when a patient develops bilateral disease [7,11]. The treatment of the patient may therefore change depending on whether they have unilateral or bilateral disease. It is important to note from our findings that the majority of patients with unilateral disease would potentially benefit from interventions aimed at preventing disease in the other, apparently normal joint. Techniques such as wedged insoles, neuromuscular exercises and gait retraining may be appropriate and further research is warranted to examine ways to protect the ‘joint at risk’.

These findings have implications for clinical practice as well as future research in osteoarthritis. Patients can be given a prognosis which may help motivate them to comply with treatments and address symptoms in the other knee appropriately. Clinicians should be aware that the presence of osteoarthritis in one knee is likely to herald a process that also involves the other side in the future. Radiographic changes develop slowly and for patients with chronic knee pain “radiographically healthy knees” may not be as healthy.

## Conclusion

Researchers need to remember to account for both knees when assessing the relationship between pain, function and structural disease. The non affected knee should not be used for comparison as a control. Assessment of any new treatment should focus on the effects on both joints, whether unilateral at the start of the study or not.

As new technologies are developed to treat knee osteoarthritis, there is significant potential benefit to be gained from preventative treatments of the ‘knee at risk’ in a patient with unilateral disease. Future interventions aiming at slowing the progression to bilateral disease may well be of benefit to the patient with osteoarthritis.

## Competing interests

The authors declare that they have no competing interests.

## Authors' contributions

CAT and MLEA were involved in the collection of the dataset as described previously [12,13], The data was analysed by AJM, MLEA, RG and CAT. All authors read and approved the final manuscript.

## Declaration of funding and role of funding source

Grants were received from The Swedish Rheumatism Association, The Gothenburg Rheumatism Association, The Norrbacka-Eugenia Foundation, and The Halland County Council. The funding sources had no role in data collection or interpretation.

## Pre-publication history

The pre-publication history for this paper can be accessed here:

http://www.biomedcentral.com/1471-2474/13/153/prepub
